# Higher Serum Insulin Concentrations Positively Influence the Bone Mineral Density in African American Adolescents

**DOI:** 10.9734/BJMMR/2013/2720

**Published:** 2013

**Authors:** Ambika P. Ashraf, Jessica Alvarez, Carrie Huisingh, Krista Casazza, Barbara Gower

**Affiliations:** 1 Department of Pediatrics/Division of Pediatric Endocrinology and Metabolism, The Children's Hospital, University of Alabama Birmingham, Birmingham, USA; 2 Division of Endocrinology, Emory University School of Medicine, Atlanta, USA; 3 Center for Clinical and Translational Sciences, University of Alabama at Birmingham, USA; 4 Department of Nutrition Sciences, University of Alabama Birmingham, Birmingham, USA

**Keywords:** *Bone mineral density*, *insulin secretion*, *ethnic differences*, *bone mass*

## Abstract

**Background:**

Puberty is a developmental stage of increased insulin resistance that also is a critical period for bone mass accrual. Historically, African Americans (AA) have lesser risk for osteoporotic fractures compared to European Americans (EA). AA also have higher incidence of insulin resistance. The possibility that bone health and insulin secretion or concentrations are linked has not been investigated.

**Aims:**

We aimed to examine the associations of bone mineral density (BMD) and bone mineral apparent density (BMAD) with insulin sensitivity and secretion in healthy adolescent girls and healthy female adults and to evaluate ethnic differences in these associations.

**Study Design:**

Observational cohort design.

**Place and Duration of the Study:**

University of Alabama at Birmingham, between January 2010 and September 2011.

**Methodology:**

Healthy, female, non-smoking adolescents and young adults (14–55 years) were enrolled in this observational cohort study.

**Results:**

Adolescents had significantly higher fasting insulin (*P*=0.0002), insulin area under the curve [AUC] (*P*= 0.0004) and lower insulin sensitivity (*P*=0.0005) compared to adults. Among adolescents, AA race was significantly associated with BMD (β=0.086, P=0.01) and BMAD (β=0.0075, P=0.002); however, adjusting for insulin AUC explained this difference. Insulin AUC (β=0.0006, P=0.029) and fasting insulin (β=0.0005, P=0.01) were positively associated with BMAD only in AA adolescents. Insulin AUC and fasting insulin were not significant predictors of BMD for adults.

**Conclusion:**

The higher insulin concentration among AA adolescents is associated with increased BMD and higher BMAD.

## 1. INTRODUCTION

Insulin is considered to be an anabolic agent of bone by many authors [1–5]. However, recent investigations suggest a potential adverse influence of diabetes and pre-diabetes on the bone [6,7]. It is also widely reported that African Americans (AA) have a greater degree of insulin resistance compared to European Americans (EA) [8], and puberty is a developmental state of increased insulin resistance [9]. Adolescence is considered to be the critical period during which the `peak bone mass' is achieved [10]. Many studies have confirmed that AA have much higher bone mineral density (BMD) and bone mineral content (BMC) than EA, beginning during childhood and adolescence [11]. Hence it is important to understand the relationships between insulin (especially insulin secretion and sensitivity) on BMC and BMD in healthy adolescents and adults and to identify if are any ethnic differences in these relationships. We hypothesized that the higher insulin concentrations will result in better bone mass and this may be more pronounced in AA adolescents due to their higher insulin concentrations. We also hypothesized that the effect of insulin will be mediated in part by the fat mass.

Osteoporosis and related fractures are known to cause considerable morbidity and financial burden [12]. Yet, even now, determinants of bone mass in adolescents and young adults are not well known. An estimated 80% of the bone mass is attributed to genetic factors, however, the remaining 20% of modifiable factors may have a crucial role in regulation of bone mass and density [13,14]. The known determinants of BMD and BMC are body weight, height, lean body mass, fat mass, sex and race [1,15–18]. Dual-energy x-ray absorptiometry (DXA) is the most widely used method for assessing BMD and is a well studied and verified surrogate measure of bone health. In healthy, normally growing children, DXA measures of BMC and BMD increase as a function of age and sexual maturation, and both increase substantially during childhood and adolescence. Low BMD is considered a manifestation of poor mineral accretion or foreboding of osteoporosis and, hence, assessment of bone health in adolescents and young adults is a major issue [11]. Therefore, the objective of the present study was to examine the ethnic differences in insulin sensitivity and secretion and to evaluate the associations of BMD with insulin secretion and sensitivity in healthy, non-diabetic, adolescent girls and healthy female adults.

## 2. MATERIALS AND METHODS

### 2.1 Subjects

Subjects were female, pre menopausal adults (18–55 years) and female adolescents (14–18 years) who were previously enrolled in 2 observational cohort studies: the VIVID Study and the DIVA Study (Clinical Trial Registration Number: NCT01041547, NCT01041365). The data were collected between January 2010 and September 2011. The Institutional Review Board of the University of Alabama at Birmingham (UAB) approved both studies. Written informed consent in all subjects and assent in those <18 years of age was obtained before entry to the study. Ethnicity (European American - EA, African American - AA) was self reported. Exclusion criteria were chronic illnesses such as diabetes, inflammatory disorders, hypertension, subjects on anti-hypertensive, glucose-controlling, lipid-lowering medications or steroids; smoking; body mass index (BMI, kg/m^2^) >95^th^ centile for age and sex according to the Centers for Disease Control and Prevention growth charts [19] for adolescents and >32 kg/m^2^ for adults and lactose intolerance, non-ambulatory subjects. Because obesity is associated with increased BMD [20], we selected subjects who are non-obese, so as not to confound with potential findings with obesity. As insulin resistance is affected by pubertal status, only adolescents who were menarchal and in Tanner stage ≥ 4 for breast and pubic hair development [21] were included in the <18 year old group. Post menopausal women were excluded from the analysis.

### 2.2 Methods

Body composition (fat and lean mass) and bone mass were assessed using DXA (iDXA, GE-LUNAR Radiation Corp., Madison, WI). Bone mass, as measured by DXA at whole body, is reported as bone mineral content (BMC) (grams) or areal BMD (BMC/bone area g/cm^2^). Whole body bone mineral apparent density (BMAD, g/cm^3^), was calculated according to the formula, BMC/[whole body mineral area^2^/body height] [22,23]. Bioelectrical impedance analysis (BIA) was used to measure participants' lean body mass using Tanita BC-418 segmental body composition analyzer (Tanita Corporation, Arlington Heights, IL).

For each subject, a mixed meal tolerance test (MMTT) was performed using Carnation Instant Breakfast prepared with whole milk in a dosage of 11.5 kcal/kg of lean body mass (LBM) [1.75 gm/kg LBM of carbohydrate], followed by repeated blood draws at baseline and at 5, 10, 15, 20, 25, 30, 45, 60, 90, 120, 150, and 180 minute after the start of meal ingestion. Plasma samples were stored at −80°C until assay.

### 2.3 Laboratory Analyses

Glucose was assayed in 10 μl sera using a Sirrus analyzer (Stanbio, Boerne, TX). The mean intra- and inter-assay coefficients of variation (c.v.) for glucose analysis in the Core Laboratory are 1.28% and 1.53%, respectively. Insulin was assayed by immunofluorescence on a TOSOH AIA-II analyzer (TOSOH Corp., South San Francisco, CA); intra-assay CV of 1.5% and inter-assay CV of 4.4%. Whole-body insulin sensitivity was calculated as proposed by Matsuda et al. [24], where WBISI = 10,000/ √ (fasting glucose × fasting insulin) × (mean glucose × mean insulin during OGTT). The homeostatic model assessment of basal insulin resistance (HOMA-IR) was calculated using the formula: HOMA-IR = [fasting insulin (μU/ml) × fasting glucose (mmol/L)] / 22.5 [25,26]. Post-MMTT area under the curve (AUC) and incremental AUC for insulin and glucose were calculated using the trapezoidal method. [27]. The serum 25(OH)D assays were obtained commercially (Quest Diagnostics, Nichols Institute, San Juan Capistrano, California), using Liquid chromatography- tandem mass spectrometry (LC- MS/MS) methodology which is considered to be the reference standard for 25(OH)D assay [28]. Serum PTH was commercially assessed by a two-site immunoradiometric assay that detects intact PTH (1- 84) and the amino- terminally truncated PTH (7–84) fragments (normal range 10–65 pg/ml) (Quest Diagnostics Nichols Institute, San Juan Capistrano, California).

### 2.4 Statistical Analyses

Demographic and body composition characteristics were compared between adults and adolescents as well as between EA and AA subjects. Chi-square (Fisher's exact when expected cell counts were <5) and Student's *t*-test were used for categorical and continuous variables, respectively. Spearman correlation coefficients were used to assess collinearity between serum 25(OH)D with the other independent variables. General linear models were used to assess the association of clinical characteristics with BMD and BMAD. Separate multivariable linear regression models were performed to adjust for potential confounders individually such as height, lean mass, percent body fat, fasting insulin, insulin AUC. P ≤ 0.05 was considered statistically significant. Analyses were performed using SAS software (version 9.2; SAS Institute, Cary, NC).

## 3. RESULTS

Table 1 illustrates the characteristics of adult and adolescent female subjects. Compared to adults, healthy adolescent females had significantly higher insulin AUC (P=0.0004), lower WBISI (P=0.0005) and higher fasting insulin (P= 0.0002). Total fat mass, percent body fat, serum 25(OH)D and BMD were not significantly different between adults and adolescents. When both cohorts were stratified by race (Table 2), there were no statistically significant differences in age, BMI, percent body fat, or Z-score between AA and EA adolescents or AA and EA adults. Serum 25(OH)D was significantly lower among AA adolescents compared to EA adolescents (P<0.0001) and among AA adults compared to EA adults (P=0.0003). There was no difference in BMD Z-score between AA and EA adolescents or adults. The mean BMAD was statistically higher among AA adolescents compared to EA adolescents (P=0.0024).

Spearman correlation coefficients between the independent variables and 25(OH)D (data not shown) demonstrate that there was a statistically significant correlation between 25(OH)D with total fat mass (rho=−0.597 and percent body fat (rho=−0.714) among EA adolescents. There were no significant correlations between the independent variables and 25(OH)D among AA adolescents, AA adults or EA adults. There was a moderately significant positive correlation between 25(OH)D with BMD (rho=0.463, P=0.071) and with BMAD (rho=0.539, P=0.031) among AA adults only. Among adolescents and adults, there were no statistically significant differences in mean BMD or BMAD between those with serum 25(OH)D concentrations of 20 ng/ml or 30 ng/ml (data not shown).

Associations of BMD and BMAD with variables for adults and adolescents are illustrated in Table 3. Among adults, body weight, BMI, and lean mass were significant predictors of BMD. There were no significant predictors of BMAD among adults. Serum 25(OH)D was not significantly associated with BMD or BMAD. Among adolescents, body weight, BMI, total fat mass, lean mass, in addition to percent body fat were significant predictors of BMD and BMAD. Similar to adults, 25(OH)D was not significantly associated with BMD or BMAD among adolescents. Among adolescents, AA race was significantly associated with BMD and BMAD. The association with BMD persisted after adjusting for percent body fat; however, adjustment for insulin AUC (adjusted β=0.012, P=0.76), fasting insulin (adjusted β=0.062, P=0.067) and lean mass (adjusted β=0.046, p=0.072) attenuated the significance of race with BMD. The association of AA race with BMAD persisted after adjusting for % body fat (adjusted β=0.0076, P=0.0008), lean mass (adjusted β=0.0059, P=0.012), and fasting insulin (adjusted β=0.0055, P=0.023), but did not persist after adjusting for insulin AUC (adjusted β=0.0027, P=0.41). Among adults, AA race was associated with BMD even after adjusting for fasting insulin (adjusted β=0.097, P=0.034) and insulin AUC (adjusted β=0.10, P=0.036). Adjusting for percent body fat (adjusted β=0.087, P=0.065) and lean mass (adjusted β=0.059, P=0.19) attenuated the significance of AA race with BMD among adults. Fasting insulin and insulin AUC were strongly associated with BMD and BMAD in adolescents. After adjusting for percent body fat, the associations with BMD remained statistically significant for fasting insulin, but not for insulin AUC. Among adolescents, BMD increased 0.0055 [units] for every unit increase in fasting insulin after adjusting for percent body fat. Fasting insulin and insulin AUC were not associated with BMD after adjusting for lean mass. The associations with fasting insulin and insulin AUC with BMAD remained significant after adjusting for percent body fat and lean mass.

Associations between BMAD with variables by race are illustrated in Table 4. Among EA adults, insulin AUC and fasting insulin were not significant predictors of BMAD. Among AA adolescents, fasting insulin (β= 0.00052, P= 0.011) and insulin AUC (β= 0.00061, P= 0.029) were significant predictors of BMAD; however this association disappeared after adjusting for percent fat. Insulin AUC and fasting insulin were not significant predictors of BMAD for AA adults (fasting insulin, β= −0.00052, P=0.66; Insulin AUC, β = 0.00011, P= 0.91), EA adults (fasting insulin, β= −0.00028, P=0.63; Insulin AUC, β = −0.000096, P= 0.92) or EA adolescents (fasting insulin, β= −0.00029, P=0.73; Insulin AUC, β =−0.00066, P= 0.53).

## 4. DISCUSSION

To our knowledge, little has been reported about the associations between insulin secretion and sensitivity and BMD and BMAD in healthy adolescents. The beneficial roles of insulin on skeletal health has been entertained by many authors [1], supported by the observations that lower BMD in type 1 diabetes [2,3] and higher BMD in subjects with type 2 diabetes [4,5]. We found that there are stronger associations of BMAD with fasting insulin and insulin AUC in AA adolescents. Insulin can exert direct effect on bone cells as it promotes osteoblasts proliferation and osteoblastogenesis and inhibits apoptosis [1]. Moreover, insulin acts synergistically with the salutory bone effects of insulin like growth factor-1 (IGF-1) and PTH [1]. Moreover, studies have alluded to a signaling pathway connecting bone and glucose metabolisms, mainly through an interconnected hormonal pathway that involves leptin, osteocalcin, and adiponectin [29]. Our study suggests that higher fasting insulin levels and insulin secretion observed in AA adolescents may be beneficial for their bone health. However, in adults, the insulin effect disappears. Therefore, it is possible that the peak bone mass in AA may be acquired during puberty in conjunction with the higher insulin.

This observation should not be taken out of the context that pre-diabetes and type 2 diabetes (T2DM) adversely influences the bone health [6,7]. Prepubertal, obese children with pre-diabetes reportedly have lower bone mass compared to obese, non-diabetic children [7]. It is not known whether the bone is relatively spared in obesity despite the cellular resistance to insulin in critical tissues such as fat, liver and muscles. Pre-diabetes and T2DM are states of both insulin resistance and impaired insulin secretion, resulting in abnormal glucose regulation; hence, the increased incidence of poor skeletal health reported in those individuals [4,7] may not reflect the independent actions of insulin on bone.

Corroborating other studies, we found that body weight, BMI, fat mass, and lean mass are associated with BMD in adolescents and young adults. The effect of insulin is mediated in part by fat mass among AA adolescents, which is suggested by the disappearance of the association between insulin measures and BMAD after adjusting for percent fat. Although total fat mass has a positive association with total bone mass, visceral adiposity measures reportedly have negative associations with bone mass [7]. We did not measure disparate adipose compartments, but found a strong positive correlation between total bone mass and percent fat in normal weight AA adolescents. When we adjusted for %fat, the association of BMD with insulin AUC and fasting insulin disappeared implying that the % fat influences the associations. We postulate that insulin affects fat deposition which in turn has beneficial effects on bone by some signaling factors such as leptin [30]. We have not assessed any mediators for this study. Similarly we have not measured subcutaneous vs. visceral adiposity to see if the association is influenced differentially by the type of fat compartment. Larger studies evaluating whether the insulin directly affects the bone, or if insulin influences fat mass, which in turn positively affects the bone will be helpful to delineate this speculation.

Strengths of the study were that we included healthy, non-obese, non-smoking, post menarchal adolescent females and adult females as our research subjects. Our study is unique in that we evaluated the insulin response to a mixed meal in a physiological fashion which allowed us to optimally delineate the insulin response to a meal. To our knowledge, this is the first study to assess relationships between meal-stimulated insulin and BMD in this particular age group. Moreover, we found that the ethnic differences in insulin secretion in the critical adolescent time period of life influence BMD and BMAD. This study is also strengthened by the inclusion of adults in addition to adolescents as it clearly demonstrates the advantages of enhanced adolescent insulin secretion on BMAD.

Despite strengths, it is not without limitations. We do not have their calcium intake data. It has been reported that bioavailable vitamin D may be more important in determining BMD and BMAD [31]. We have not assessed other factors that may potentially play a role such as free (unbound) vitamin D and IGF-1. We have not utilized peripheral quantitative computed tomography (pQCT) and hence cannot ascertain whether the better BMD in AA adolescents translates to better bone quality and microarchitectural integrity. Our study was limited by the small sample size particularly among the adult female cohort, which makes our findings less generalizable.

## 5. CONCLUSIONS

Our data demonstrate that in non-obese, non-diabetic African American adolescents, insulin secretion of puberty is associated with greater BMD; however, the extent to which the greater BMD is beneficial to skeletal health warrants investigation.

## Figures and Tables

**Table 1:** Descriptive, metabolic and bone mineral characteristics

	Adults (N=29)	Adolescents (N=49)	
Variable	Mean± SD or n (%)	Mean± SD or n (%)	P
Age (yr)	31.7± 8.8	15.7± 1.4	<0.0001
Race: AA/EA	16 (55.2)/ 13 (44.8)	33 (67.3)/ 16 (32.7)	
Body weight (kg)	66.9± 14.0	62.7± 12.2	0.18
Height (cm)	165.6± 6.5	164.7± 7.0	0.59
BMI (kg/m^2^)	24.3± 4.2	23.1± 4.0	0.22
Region percent body fat (%)	32.4± 7.4	29.7 ± 5.9	0.070
Lean mass (kg)	41.7± 5.3	41.03 ± 6.0	0.61
25(OH)D (ng/ml)	23.3± 10.7	19.3 ± 8.6	0.079
PTH (pg/ml)^1,2^	--	36.5± 14.6	--
Bone mineral content (gm)	2543.2± 380.3	2471.2± 349.2	0.40
Bone mineral density (g/cm^2^)	1.2± 0.1	1.2± 0.1	0.15
Whole body bone mineral apparent density (g/cm^3^)	0.095 ± 0.010	0.091± 0.008	0.0467
Z-score	0.63± 1.1	0.7±1.0	0.89
Insulin AUC (ulU/ml)	6293.6± 2775.5	11,619.3± 6,174.2	0.0004
WBISI	10.2± 5.6	5.6± 3.1	0.0005
Fasting insulin (ulU/ml)	5.4± 2.9	9.4± 5.8	0.0002

**Legend:** Reported as mean ± S.D or n (%).

Abbreviations: AA, African American; EA, European American; BMI, body mass index; 25(OH)D, 25-hydroxyvitamin D; PTH, parathyroid hormone; AUC, area under the curve; WBISI, whole body insulin sensitivity index.

Methods: Student's t-test and chi-square test were used to determine statistical significance.

1 n=29 missing from adults,

2 n=16 missing from adolescents

**Table 2:** Variable characteristics by race and age

Variable	Adults Mean ± SD	P Value	Adolescents Mean ± SD	P Value
Age (yr)	AA: 32.9±10.5	0.43	15.5±1.4	0.12
	EA: 30.2±6.3		16.2±1.4	
Body weight (kg)	AA: 72.4±14.6	**0.016**	64.3±13.1	0.20
	EA: 60.1±10.2		59.5±9.6	
BMI (kg/m^2^)	AA: 25.3±4.5	0.14	23.8±3.8	0.077
	EA: 23.0±3.5		21.6±3.9	
Height (cm)	AA: 168.8±6.0	**0.0016**	164.0±7.5	0.30
	EA: 161.6±4.7		166.2±5.7	
Percent body fat (%)	AA: 34.5±7.5	0.10	29.6±5.6	0.89
	EA: 29.9±6.8		29.8±6.2	
Lean mass (kg)	AA: 43.7±5.7	**0.026**	42.1±6.5	0.076
	EA: 39.3±3.8		38.9±4.3	
25(OH)D (ng/ml)	AA: 16.8±4.8	**0.0003**	14.9±5.5	<**0.0001**
	EA: 31.3±10.7		27.9±6.7	
PTH (pg/ml)	AA —	--	37.0±13.8^1^	0.76
	EA --		35.2±17.4^2^	
Bone mineral content (gm)	AA: 2718.6±334.6	**0.0038**	2516.9±387.5	0.21
	EA: 2327.5±325.5		2382.5±247.0	
Bone mineral density (g/cm^2^)	AA: 1.3±0.1	**0.031**	1.2±0.1	**0.011**
	EA: 1.2±0.1		1.1±0.1	
Whole body bone mineral apparent density (g/cm^3^)	AA: 0.097±0.011	0.15	0.094±0.008	**0.0024**
	EA: 0.092±0.0006		0.086±0.007	
Z-score	AA: 0.4±1.3	0.19	0.8±1.1	0.30
	EA: 0.9±0.8		0.4±0.9	
Insulin AUC (ulU/ml)	AA: 6958.6±3124.1	0.16	14223.9±6165.9	0.0004
	EA: 5475.1±2111.6		6988.8±2320.6	
WBISI	AA: 8.8±3.3	0.18	4.1±1.9	0.0002
	EA: 11.8±7.4		8.4±2.8	
Fasting insulin (ulU/ml)	AA: 5.6±2.6	0.79	10.7±6.6	0.0027
	EA: 5.3±3.4		6.6±2.3	

**Legend:** Reported as mean ± S.D or n (%).

Abbreviations: BMI, body mass index; 25(OH)D, 25-hydroxyvitamin D; PTH, parathyroid hormone; AUC, area under the curve; WBISI, whole body insulin sensitivity index.

Methods: Student's t-test and chi-square test were used to determine statistical significance.

1 n=9 missing from AA adolescents,

2 n=7 missing from EA adolescents

**Table 3:** Associations of BMD and BMAD with outcome variables

	Variable	Adults		Adolescents	
		β	*P*	β	*P*
BMD	Race (AA vs. EA (ref))	0.097	**0.031**	0.086	**0.011**
	Body weight	0.0038	**0.019**	0.0066	<**.0001**
	BMI	0.012	**0.029**	0.018	<**.0001**
	Total fat mass	0.0045	0.068	0.0098	<**.0001**
	Percent body fat	0.0040	0.20	0.0084	**0.0023**
	Lean mass	0.011	**0.0083**	0.013	<**.0001**
	25(OH)D	−0.0022	0.32	−0.0036	0.062
	Insulin AUC	0.0000032	0.70	0.0064	**0.016**
	WBISI	−0.0014	0.74	−0.0053	0.36
	Fasting insulin	0.00090	0.91	0.0074	**0.0065**
WB BMAD	Race (AA vs. EA (ref))	0.0050	0.17	0.0075	**0.0024**
	Body weight	0.000060	0.65	0.00035	**0.0002**
	BMI	0.00032	0.47	0.0010	**0.0005**
	Total fat mass (kg)	0.000061	0.77	0.00057	**0.0004**
	Percent body fat	0.000032	0.90	0.00054	**0.0089**
	Lean mass (kg)	0.00017	0.62	0.00061	**0.0019**
	25(OH)D	−0.000042	0.81	−0.00025	0.081
	Insulin AUC (per 1000)	0.00030	0.66	0.00064	**0.0046**
	WBISI	0.00010	0.76	−0.00075	0.13
	Fasting insulin	−0.00034	0.60	0.00062	**0.0018**

Legend: The crude β coefficients (change in BMD/BMAD for every unit change in the select variable) and P-vaiues from the general linear model.

Abbreviations: BMI, body mass index; 25(OH)D, 25-hydroxyvitamin D; BMD: bone mineral density, BMAD: bone mineral apparent density, Insulin AUC: insulin area under the curve, WBISI: whole body insulin sensitivity index

**Table 4:** Analyses of associations by race and age

		European American Adolescents n=16 Adults n=13		African American Adolescents n=33 Adults n=16	

	Variable	β	*P*	β	*P*
**WB BMAD Adolescents**	Body weight	0.00021	0.30	0.00033	0.0006
	BMI	0.00030	0.54	0.0011	0.0010
	Region percent body fat	0.000044	0.89	0.00086	0.0001
	Lean mass (kg)	0.00059	0.18	0.00046	0.030
	25(OH)D	0.000094	0.75	0.00015	0.58
	Insulin AUC (per 1,000)	−0.00066	0.53	0.00061	0.029
	WBISI	0.0012	0.13	−0.0016	0.087
	Fasting insulin	−0.00029	0.73	0.00052	0.011
**WB BMAD Adults**	Body weight	0.00020	0.28	−0.00011	0.59
	BMI	0.00071	0.18	−0.00010	0.88
	Region percent body fat	0.00018	0.54	−0.00025	0.54
	Lean mass (kg)	0.00047	0.35	−0.00021	0.70
	25(OH)D	0.0000080	0.97	0.0011	0.076
	Insulin AUC (per 1,000)	−0.000096	0.92	0.00011	0.91
	WBISI	0.00018	0.49	0.00051	0.59
	Fasting insulin	−0.00028	0.63	−0.00052	0.66

Legend:

Abbreviations: BMI, body mass index; 25(OH)D, 25-hydroxyvitamin D; Insulin AUC: insulin area under the curve, WBISI: whole body insulin sensitivity index; WB BMAD- whole body apparent bone mineral density.
